# Editorial: Functional genomic approaches in molecular breeding for crop improvement

**DOI:** 10.3389/fgene.2023.1301501

**Published:** 2023-10-26

**Authors:** Sabrina M. Elias, Richard Malo, Zeba I. Seraj, Philomin Juliana

**Affiliations:** ^1^ Department of Life Sciences, Independent University Bangladesh, Dhaka, Bangladesh; ^2^ Overseas Marketing Corporation Private Ltd., Panthapath, Bangladesh; ^3^ Department of Biochemistry and Molecular Biology, University of Dhaka, Dhaka, Bangladesh; ^4^ International Maize and Wheat Improvement Center, Texcoco, Mexico

**Keywords:** QTL, expression, DNA marker, genomic selection, molecular breeding

The Research Topic has collated contributions from experts working in quantitative genomics with the integration of genetics, genomics, transcriptomics, metabolomics and phenomics. Thinking beyond conventional breeding approaches, this multi-omics approach helped gather information for enhancement of certain traits such as yield, disease resistance, secondary metabolite content, and mineral content, along with many others. This combined report comprises original research and review articles focusing improvement in Brassica, wheat, rice, soybean, and a medicinal plant. There is information on novel technologies for genotyping, genomic selection, expression, and metabolome analysis for identification and candidate gene validation through functional analysis.

The seed size of oilseed crops is of great importance for improving seed yield and quality, hence Mathur et al. performed a pairwise comparison of differentially expressed genes between two lines of *B. juncea*, EH-2 with small seeds and PJ with large seeds. The authors could identify 954 differentially expressed genes belonging to various families of transcription factors. Moreover, the DEG and co-expression datasets were consolidated with the thousand seed weight QTL, mapped earlier in a doubled haploid population of *B. juncea*. Such amalgamation allowed the authors to identify 40 potential key components controlling seed size. The promising elements were mostly involved in cell cycle, cell wall biogenesis/modification, solute/sugar transport, and hormone signaling. Transcriptional trajectories at the early stages of seed development are mostly responsible for the determination of final seed size by restricting cell numbers.

Mallick et al. reported the development of a Near Isogenic Line (NIL) in wheat HD2932, which is both leaf- and stripe rust–resistant. This fungal pathogen–resistant, closely related genotype can be utilized in future wheat breeding programs. The leaf rust–resistant gene LrTrk and the stripe rust–resistant gene YrTrk were identified in the durum wheat genotype Trinakria and transferred to HD2932. This co-introgression successfully produced the NIL lines with leaf and stripe rust resistance genes LrTrk/YrTrk.

From Brassica and wheat, the Research Topic deals with the source of the traditional Chinese medicine Radix Astragali, which is used to clinically treat respiratory, cardiovascular, immunological, and hepatic diseases. This medication is the source of a membranous flavonoid and a regulator. Hu et al. analyzed the association between flavonoid content and gene expression pattern in six different fruit developmental stages and found that the expression levels of genes involved in the phenylpropane pathway were higher than those of genes involved in the flavonoid biosynthesis pathway. Gene expression trends were classified into different profiles, followed by the identification of positive and negative correlations between the gene expression pattern and flavonoid content. In addition to understanding the flavonoid synthesis mechanism, this will pave the way for the development of high flavonoid rich molecular breeding in *Astragalus membranaceus*, the source of Radix Astragali.

The review article by Roy et al. compiled existing knowledge on the physiological and molecular mechanisms of zinc and iron uptake, translocation, and grain sequestration. It also provided information about mapping of genes and QTL and new breeding techniques that need to be adopted for biofortification of staple food crops like wheat. There were studies where negative associations were found between Zn and Fe and grain yield. Hence, the authors recommended that biofortification can be achieved with mineral uptake enhancement and deposition into grain, ensuring the bioavailability of mineral uptake and the source to sink translocation of minerals. Integration of genomic selection, speed breeding, and genome editing was endorsed.

Transcriptomic and metabolomic analyses by Chu et al. identified differentially expressed genes and metabolites in PI88788 type and Peking type cultivars of Soybean. Previously, Rhg1 and Rhg4 were identified as two QTLs playing the most important roles in conferring resistance against Soybean Cyst Nematode (SCN), which is a widely occurring pathogen. Soybean accessions identical to Peking and PI88788 at these loci were selected and classified into two separate groups to reveal the different mechanisms of resistance in these two varieties since Peking- and PI 88788-type sources showed different responses to SCN infections. It was predicted that the metabolism of toxic compounds played a key role in conferring soybean cyst nematode resistance to soybean. GmLOX1 (Glyma.15G026500) was commonly upregulated in both Peking- and PI 88788- type sources, and was also identified by integrative analyses of transcriptome and metabolome shown to be involved in Jasmonic Acid biosynthesis. Their results showed that GmLOX1 overexpression could enhance resistance to SCN in transgenic hairy roots, confirming the role of JA in the response to SCN infection. Moreover, overexpression of GmERF71 decreased the number of cysts by 59% compared with the control, confirming its function in conferring resistance.

The last article by Biswas et al. is an example of the practical application of genomic selection (GS) in the field. Along with capturing high-value parents, GS technology can predict the performance of selected tested and untested lines, obviating the need for more extensive analysis of the whole population. However, the precision of prediction depends on the model accuracy, crop size, trait heritability, the extent of linkage disequilibrium, and the size of the training population. In addition, there is a requirement for multiple environmental and yearly trials in order to account for the interaction between different factors. The study reports how applying GS and trait specific marker–assisted selection together expedited the improvement in rice yield to 117 kg ha^-1^year^-1^, which is approximately 7-fold higher than current levels. Moreover, there was a shortening of the breeding cycle by approximately 1.5 years from the existing 4.5 years.

We hope that readers will find this Research Topic interesting and a useful reference for state-of-the-art technology. The theme exemplifies the emerging field of the use of tools rooted in the blending of variability in the genome, transcriptome, metabolome, and phenome, followed by functional analysis of the candidate genes.

